# Supplementary material for: Increasing number of long-lived ancestors marks a decade of healthspan extension and healthier metabolomics profiles
Source: Nat Commun. 2023 Jul 27;14:4518. doi: 10.1038/s41467-023-40245-6 (PMC10374564; doi:10.1038/s41467-023-40245-6)
Supplement: Supplementary file 1 — Supplementary Information [file 41467_2023_40245_MOESM1_ESM.pdf]

Supplementary Table 1: Time from study inclusion to first disease and multimorbidity

	A: Time from inclusion to first disease				B: Time from inclusion to 2 diseases			
	N (prop)	Events (prop)	HR (95% CI)	P-Value	N (prop)	Events (prop)	HR (95% CI)	P-Value
**Age related diseases**								
Groups								
Offspring	917 (0.70)	362 (0.39)	0.79 (0.65-0.97)	$2.32 \cdot 10^{-2}$	611 (0.70)	73 (0.12)	0.55 (0.36-0.85)	$6.83 \cdot 10^{-3}$
Partners (ref)	395 (0.30)	171 (0.43)			268 (0.30)	47 (0.18)		
Sex								
Females	733 (0.56)		0.79 (0.66-0.94)	$8.78 \cdot 10^{-3}$	611 (0.70)		0.59 (0.40-0.89)	$1.04 \cdot 10^{-2}$
males (ref)	579 (0.44)				268 (0.30)			
**Metabolic diseases**								
Groups								
Offspring	917 (0.70)	261 (0.23)	0.71 (0.55-0.90)	$5.18 \cdot 10^{-3}$	611 (0.70)	45 (0.07)	0.51 (0.26-0.97)	$3.96 \cdot 10^{-2}$
Partners (ref)	395 (0.30)	135 (0.34)			268 (0.30)	29 (0.11)		
Sex								
Females	733 (0.56)		0.84 (0.68-1.05)	$1.26 \cdot 10^{-1}$	611 (0.70)		0.52 (0.30-0.92)	$2.41 \cdot 10^{-2}$
Males (ref)	579 (0.44)				268 (0.30)			
**Malignancies**								
Groups								
Offspring	917 (0.70)	130 (0.14)	0.95 (0.70-1.31)	$7.66 \cdot 10^{-1}$	611 (0.70)	7 (0.01)	1.39 (0.29-6.70)	$6.82 \cdot 10^{-1}$
Partners (ref)	395 (0.30)	56 (0.14)			268 (0.30)	2 (0.01)		
Sex								
Females	733 (0.56)		0.66 (0.49-0.88)	$4.70 \cdot 10^{-3}$	611 (0.70)		1.06 (0.28-3.96)	$9.30 \cdot 10^{-1}$
Males (ref)	579 (0.44)				268 (0.30)			

Table shows the time from inclusion to first disease in panel A, and the time from inclusion to having 2 diseases (panel B). N is the group size used for the analyses and prop. Is the proportion from the total. Events are the events of the specific diseases, for example age-related diseases, and prop. indicates the proportion from the size of a specific group (LLS IPs or partners). HR is the abbreviation for Hazard Ratio and represents the estimated effect size. Statistical testing was performed using Wald tests for the conditional log-hazard ratio estimated with a Cox-type frailty regression model. The analyses are adjusted for sex, different ages of study entry (left truncation), and right censoring. Survival curve details can be found in Supplementary Figure 3 and 4. To study time from inclusion to first disease, only persons without any disease at inclusion were studied. Note that the numbers in panel B are lower than those in panel A. This is because the censoring group reflects those for whom we have not observed any disease at the end of follow-up. As a results, persons with only one disease are excluded from the analyses.

Supplementary Table 2: Time from first disease to multimorbidity

	Time from first disease to second disease			
	**N (mean/prop)**	**Events (prop)**	**HR (95% CI)**	**P-Value**
**Age related → age related diseases**				
Groups				
Offspring	500 (0.68)	79 (0.16)	0.46 (0.26-0.83)	$9.82 \cdot 10^{-3}$
Partners (ref)	237 (0.32)	55 (0.23)		
Sex				
Females	345 (0.47)		0.45 (0.27-0.73)	$1.19 \cdot 10^{-3}$
males (ref)	392 (0.53)			
Age at inclusion	737 (60)		0.92 (0.86-0.96)	$3.49 \cdot 10^{-3}$
**Age related → Metabolic diseases**				
Groups				
Offspring	500 (0.68)	62 (0.12)	0.33 (0.14-0.81)	$1.46 \cdot 10^{-2}$
Partners (ref)	237 (0.32)	44 (0.19)		
Sex				
Females	345 (0.47)		0.41 (0.21-0.79)	$7.47 \cdot 10^{-3}$
Males (ref)	392 (0.53)			
Age at inclusion	737 (60)		0.89 (0.82-0.96)	$3.92 \cdot 10^{-3}$
**Age related → Malignancies**				
Groups				
Offspring	500 (0.68)	17 (0.03)	0.58 (0.27-1.25)	$1.66 \cdot 10^{-1}$
Partners (ref)	237 (0.32)	11 (0.05)		
Sex				
Females	345 (0.47)		0.58 (0.26-1.28)	$1.74 \cdot 10^{-1}$
Males (ref)	392 (0.53)			
Age at inclusion	737 (60)		0.89 (0.79-0.99)	$4.02 \cdot 10^{-2}$

Table shows the time from first to a specific second disease. N is the group size and prop. Events are the events of the specific disease, for example age related diseases. Mean/prop indicates a proportion for categorical measurements and the group average for continuous measurements. HR is the abbreviation for Hazard Ratio and represents the estimated effect size. Statistical testing was performed using Wald tests for the conditional log-hazard ratio estimated with a frailty Cox-type frailty regression model. The analyses are adjusted for sex and age at inclusion.

Supplementary Table 3: MetaboHealth score differences for LRC groups at LLS study inclusion

	N (mean/prop)	Beta estimate (95% CI)	P- Value
Groups			
LRC_g1: IPs with LRC ≥ 0.60	121 (0.06)	-0.098 (-0.184 / -0.012)	$2.66 \cdot 10^{-2}$
LRC_g2: IPs with LRC ≥0.1 & <0.60	1297 (0.67)	-0.032 (-0.077 / 0.012)	$1.55 \cdot 10^{-1}$
LRC_g3: Partners with LRC > 0	135 (0.07)	-0.016 (-0.091 / 0.058)	$6.61 \cdot 10^{-1}$
LRC_g4: partners with LRC = 0 (ref)	397 (0.20)		
Sex			
Females	1083 (0.56)	0.004 (-0.039 / 0.029)	$7.76 \cdot 10^{-1}$
Males (ref)	867 (0.44)		
Age at inclusion	1950 (59.20)	0.007 (0.005 / 0.010)	$1.37 \cdot 10^{-7}$
Medication use			
Yes	980 (0.50)	0.130 (0.096 / 0.164)	$1.36 \cdot 10^{-13}$
No (ref)	970 (0.50)		

In this table the metabolome of the LRC groups are compared at the moment of study inclusion using the MetaboHealth score. N indicates the total group size. Mean/prop indicates a proportion for categorical measurements and the group average for continuous measurements. Beta estimate indicates the beta coefficient of the linear regression analysis and represents the estimated effect size. Statistical testing was performed using T-tests for the Beta coefficients estimated with a mixed-model linear regression analysis. The analyses are adjusted for sex, age at inclusion, and medication use.

Supplementary Table 4: Disease groups based on ICD scores for GP data

Diseases (ICD10-code)	Age related	Metabolic	Malignancies
TIA (I60-I69)	X	X	
CVA (I63)	X	X	
AP (I20)	X	X	
MI (I21)	X	X	
Hypertension (I10)	X	X	
Diabetes (E10–E14)	X	X	
Prostate (C61)	X		X
Mamma (C50)	X		X
Lung (C34)	X		X
Colon (C18-C20)	X		X
Other (C00-D48)	X		X

Table provides an overview of the diseases, their ICD10 codes, and the disease clusters. Diseases were clustered into 3 groups based on the International Statistical Classification of Diseases and Related Health Problems (ICD-10) codes, (1) metabolic diseases, (2) malignant diseases, and (3) age related diseases, which are the combination of metabolic and malignant diseases. To increase the number of observations and therefore statistical power and because our focus is on age-related diseases (all diseases have a known association with aging) the combined age-related disease category was constructed.

Supplementary Table 5: Inclusion and exclusion criteria of the SEDD sample group

	**Number of unique remaining IDs**	**Number of unique IDs removed**
All persons in SEDD and national registers	920,195	
Parents are known (parent ids are known)	369,313	-550,882
At least one set of grandparents known (maternal or paternal ids are known)	180,769	-188,544
Observed in the years 1990-1995	121,967	-58,802
Age 45-60 in the years 1990-1995	4,831	-117,136
Parents from extinct cohort (born < 1915)	2,732	-2,099
Removal of individuals who are inpatients in 1989	2,500	-232
Other data cleaning		-3
Total number of Index Persons	2,497	

Table provides an overview of the SEDD inclusion and exclusion criteria.

Supplementary Figure 1: LLS study design

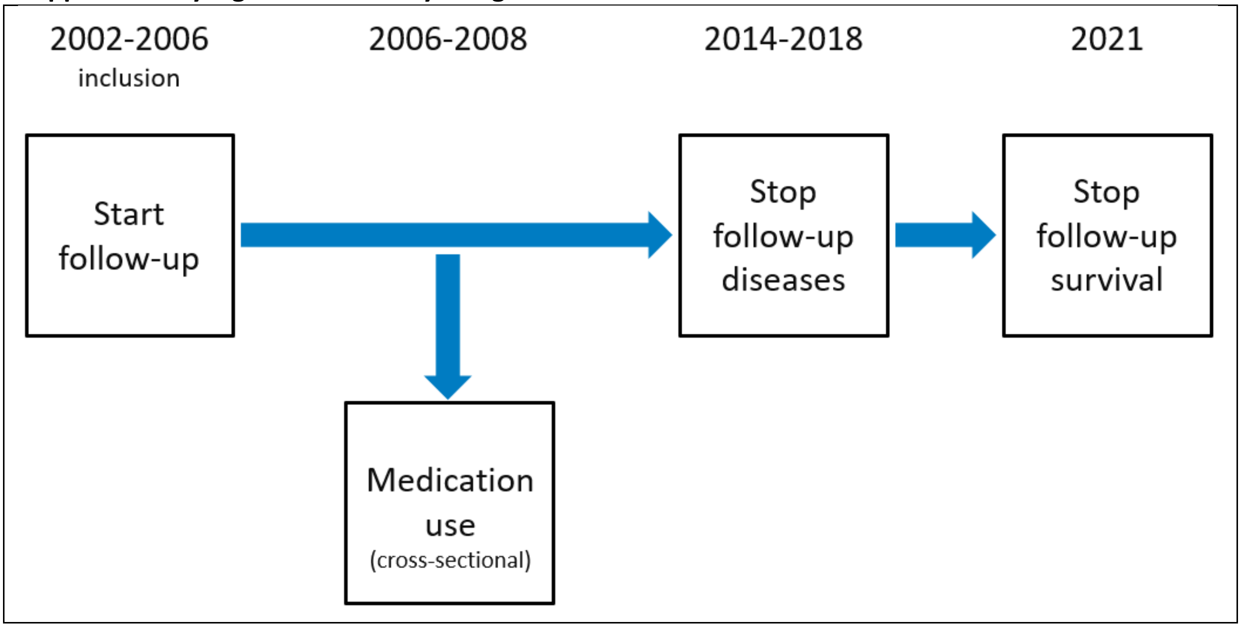

This figure illustrates the Leiden Longevity Study (LLS) design. The LLS was initiated between 2002-2006. Subsequently data on medication use was available for the period 2006-2008. Disease incidence data was available from follow-up until 2018 and mortality data was available from follow-up until 2021.

Supplementary Figure 2: Survival curves corresponding to Table 2A

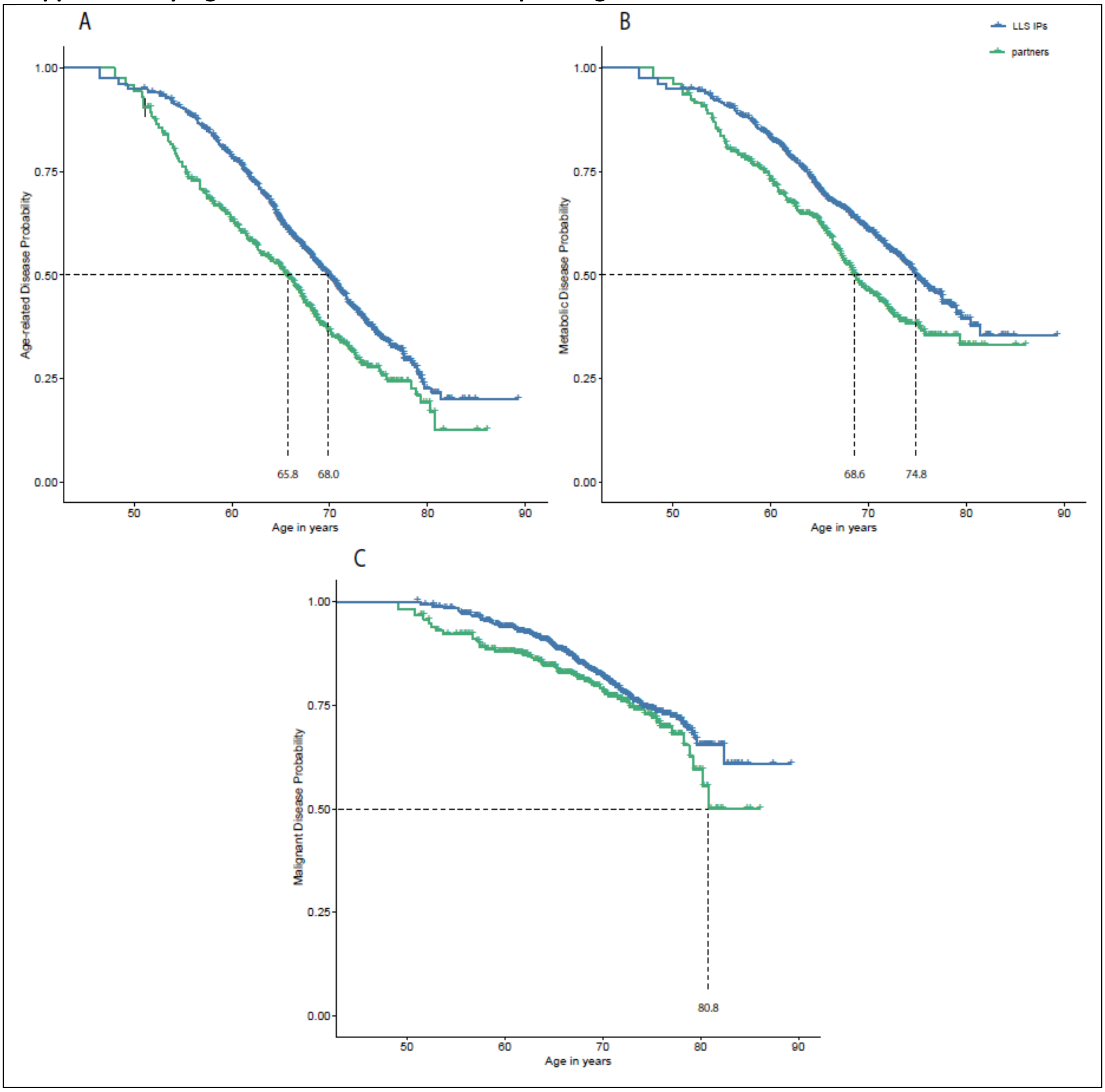

This figure depicts survival curves reflecting age-related (Panel A), metabolic (Panel B), and malignant (Panel C) disease incidence within the Leiden Longevity Study (LLS). The x-axis show age in years and the y-axis show disease incidence. Dotted lines represent the age at which 50% of the members of a specific group had their first disease. The blue line represents LLS Index Persons (IPs) and the green line represents their partners. Vertical lines within the colored lines represent right censoring events. Survival curves are adjusted for left truncation and right censoring. Source data are provided as a Source Data file.

Supplementary Figure 3: Survival curves corresponding to Table 2B

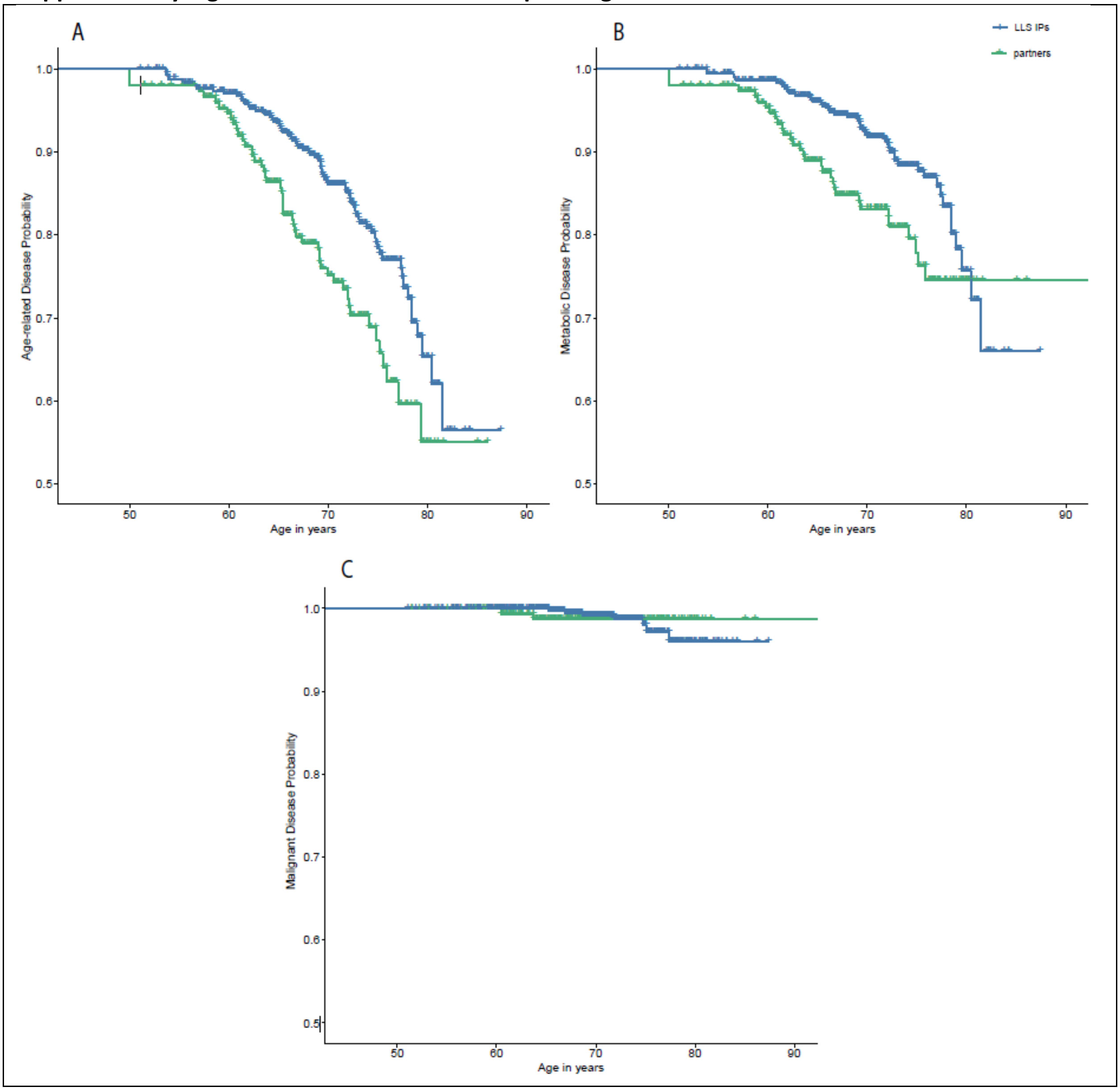

This figure depicts survival curves reflecting age-related (Panel A), metabolic (Panel B), and malignant (Panel C) disease incidence within the Leiden Longevity Study (LLS). The x-axis show age in years and the y-axis show disease incidence. Dotted lines represent the age at which 50% of the members of a specific group had 2 diseases. The blue line represents LLS Index Persons (IPs) and the green line represents their partners. Vertical lines within the colored lines represent right censoring events. Survival curves are adjusted for left truncation and right censoring. Source data are provided as a Source Data file.

Supplementary Figure 4: Survival curves corresponding to Table 2C

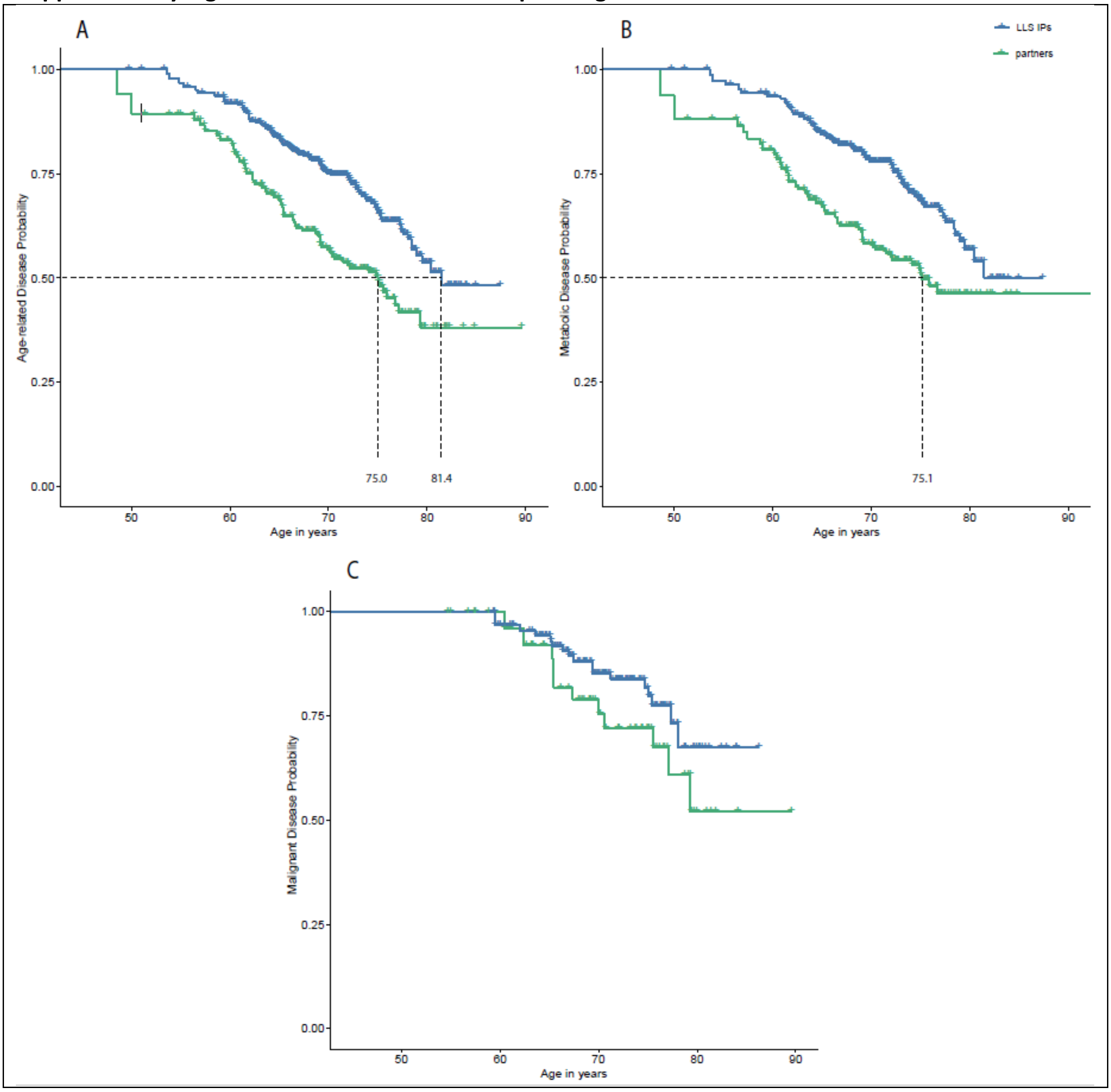

This figure depicts survival curves reflecting age-related (Panel A), metabolic (Panel B), and malignant (Panel C) disease incidence within the Leiden Longevity Study (LLS). The x-axis show age in years and the y-axis show disease incidence. Dotted lines represent the age at which 50% of the members of a specific group had their second disease after already being diagnosed with a first disease. The blue line represents LLS Index Persons (IPs) and the green line represents their partners. Vertical lines within the colored lines represent right censoring events. Survival curves are adjusted for left truncation and right censoring. Source data are provided as a Source Data file.

Supplementary Figure 5: Survival curves corresponding to Table 3

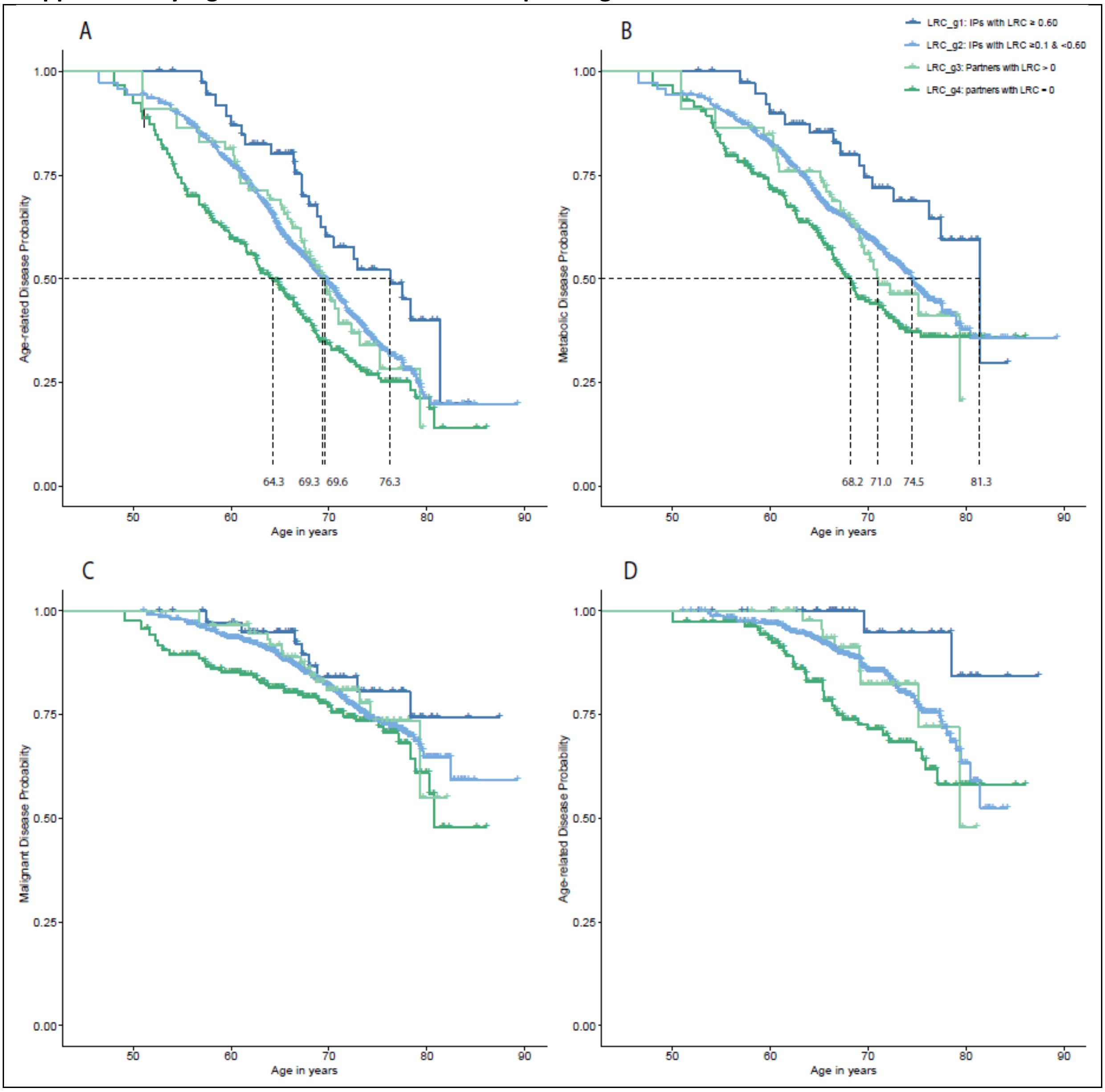

This figure depicts survival curves reflecting age-related (Panel A), metabolic (Panel B), malignant (Panel C), and age-related (Panel D) disease incidence within the Leiden Longevity Study (LLS). The x-axis show age in years and the y-axis show disease incidence. Dotted lines represent the age at which 50% of the members of a specific group had their first disease (Panel A-C) and the age at which 50% of the members of a specific group had 2 diseases. The blue line represents LLS Index Persons (IPs) and the green line represents their partners. Vertical lines within the colored lines represent right censoring events. Survival curves are adjusted for left truncation and right censoring. Source data are provided as a Source Data file.

Supplementary Figure 6: Distribution of LRC and MetaboHealth scores

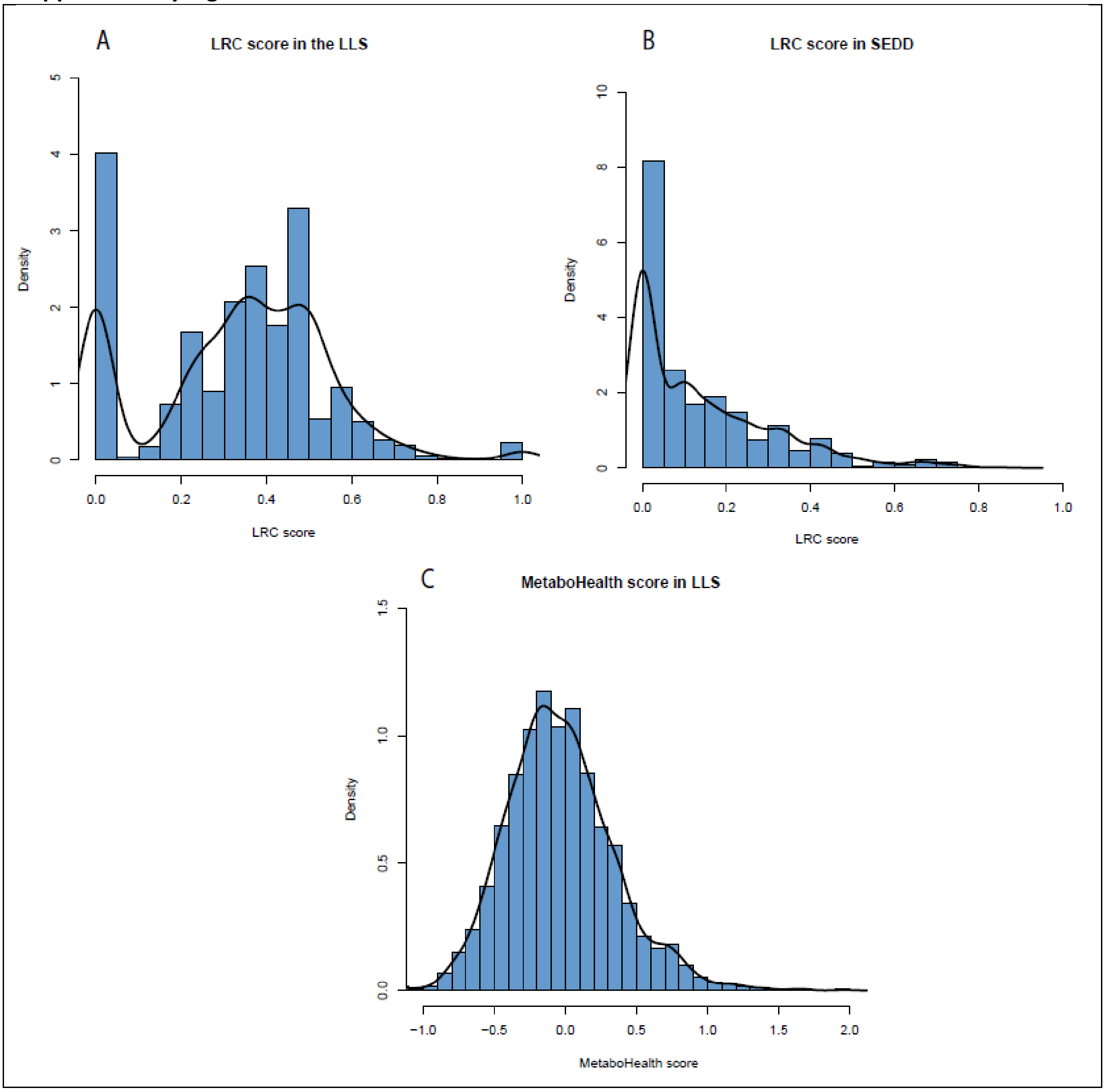

Figure depicts the distribution of the LRC score in the LLS (Panel A) and SEDD (Panel B). It further depicts the distribution of the MetaboHealth score in the LLS (Panel C). Source data are provided as a Source Data file.
