# Supplementary material for: Increasing number of long-lived ancestors marks a decade of healthspan extension and healthier metabolomics profiles
Source: Nat Commun. 2023 Jul 27;14:4518. doi: 10.1038/s41467-023-40245-6 (PMC10374564; doi:10.1038/s41467-023-40245-6)
Supplement: Supplementary file 3 — Reporting Summary [file 41467_2023_40245_MOESM3_ESM.pdf]

nature portfolio

Corresponding author(s): Niels van den Berg, n.m.a.van_den_berg@lumc.nl

Last updated by author(s): Jun 1, 2023

Reporting Summary

Nature Portfolio wishes to improve the reproducibility of the work that we publish. This form provides structure for consistency and transparency in reporting. For further information on Nature Portfolio policies, see our Editorial Policies and the Editorial Policy Checklist.

Statistics

For all statistical analyses, confirm that the following items are present in the figure legend, table legend, main text, or Methods section.

n/a	Confirmed	
☐	☒	The exact sample size (*n*) for each experimental group/condition, given as a discrete number and unit of measurement
☐	☒	A statement on whether measurements were taken from distinct samples or whether the same sample was measured repeatedly
☐	☒	The statistical test(s) used AND whether they are one- or two-sided<br>*Only common tests should be described solely by name; describe more complex techniques in the Methods section.*
☐	☒	A description of all covariates tested
☐	☒	A description of any assumptions or corrections, such as tests of normality and adjustment for multiple comparisons
☐	☒	A full description of the statistical parameters including central tendency (e.g. means) or other basic estimates (e.g. regression coefficient) AND variation (e.g. standard deviation) or associated estimates of uncertainty (e.g. confidence intervals)
☐	☒	For null hypothesis testing, the test statistic (e.g. *F*, *t*, *r*) with confidence intervals, effect sizes, degrees of freedom and *P* value noted<br>*Give P values as exact values whenever suitable.*
☒	☐	For Bayesian analysis, information on the choice of priors and Markov chain Monte Carlo settings
☒	☐	For hierarchical and complex designs, identification of the appropriate level for tests and full reporting of outcomes
☐	☒	Estimates of effect sizes (e.g. Cohen's *d*, Pearson's *r*), indicating how they were calculated

Our web collection on statistics for biologists contains articles on many of the points above.

Software and code

Policy information about availability of computer code

Data collection	For this study we use two existing datasets were used; the Leiden Longevity Study and the SEDD which was connected to the Swedish register data. We used R version 4.0.2 for the pre-processing of the data. The pre-processing code can be found in the scripts repository: https://git.lumc.nl/publications/longevity-family-diseases.
Data analysis	Data analysis was done using R version 4.0.2. The data analysis code can be found in the scripts repository: https://git.lumc.nl/publications/longevity-family-diseases.

For manuscripts utilizing custom algorithms or software that are central to the research but not yet described in published literature, software must be made available to editors and reviewers. We strongly encourage code deposition in a community repository (e.g. GitHub). See the Nature Portfolio guidelines for submitting code & software for further information.

Data

Policy information about availability of data

All manuscripts must include a data availability statement. This statement should provide the following information, where applicable:

- Accession codes, unique identifiers, or web links for publicly available datasets
- A description of any restrictions on data availability
- For clinical datasets or third party data, please ensure that the statement adheres to our policy

The individual-level data from the SEDD, the Statistics Sweden, and LLS are protected by Swedish and Dutch personal integrity laws, and other (privacy) regulations. As such, restrictions apply to the availability of the LLS and SEDD data, which were used under license for the current study, and so are not publicly available. For

both datasets, summary statistics are available upon request to the corresponding author (Niels van den Berg; corresponding author). All summary statistics and data, underlying the main figures, are available in the source data file. For both datasets, additional summary statistics are available upon request to Niels van den Berg (corresponding author; n.m.a.van_den_berg@lumc.nl). The LLS data is available for replication purposes upon reasonable request to P. Eline Slagboom (p.slagboom@lumc.nl) and if replication is conducted within the secure LUMC Leiden University Medical Center network environment. Researchers can gain access to the SEDD data as used in this study if relevant permissions have been obtained in accordance with the restrictions stated by the Regional Ethical Review Board, the Swedish Data Inspection Board, and Lund University (ingrid.van_dijk@ekh.lu.se). Initial responses for both LLS and SEDD will be within one week.

Human research participants

Policy information about studies involving human research participants and Sex and Gender in Research.

Reporting on sex and gender

Sex is considered in the study design. In table 1 we report the sex distributions in both the LLS and SEDD index persons (IPs). Table 1 shows that in all groups (IPs, parents, aunts/uncles, grandparents, and partners) sex is roughly equally distributed (~50% males and ~50% females). Sex was included in all statistical models, in accordance to our earlier work. All analyses are adjusted for sex and estimates are reported in the supplementary Tables.

In the IP's ancestors, we take maternal and paternal differences in longevity are into account by using birth cohort and sex based survival percentiles to define longevity. For example, a male born in the year 1900 belongs to the top 10% survivors of his birth cohort if he reaches an age of 86 years. A female born in 1900 belongs to the top 10% survivors if she reaches an age of 90 years. In a series of publications we showed that, using this approach we showed that the heritability/intergenerational transmission of longevity does not differ between males and females.

In the LLS, sex was first self-reported and subsequently verified with birth certificates and genotype data. Sex swaps were verified and corrected when necessary. in the SEDD, sex was obtained from the national register data and birth certificates from the ancestral data.

Population characteristics

LLS IPs and their partners, serving as environment-matched controls, were included between 2002 and 2006 at the average age of 59 years. The study inclusion was based on nonagenarian siblings in the F2 generation. Hence, IPs (F3) were included if they had at least one long-lived F2 parent and F2 aunt or uncle (females ≥ 91 years and males ≥ 89 years). The LLS consist of 651 three-generational families, defined by IP siblings who have the same parents (mean sibship size is 2.58). From inclusion onward, the IPs and their partners were followed over time, with a maximum mortality follow-up of 19 years (2002-2021) and maximum morbidity follow-up of 16 years (2002-2018). In 2021, 227 (14%) IPs and 113 (15%) partners were deceased and 1409 (84%) IPs and 619 (83%) partners were still alive. In 2018, 671 (40%) IPs and 324 (43%) partners had a disease diagnosis whereas 535 (32%) IPs and 206 (28%) partners did not have a disease diagnosis (Figure 1A and Table 1A).

The SEDD consist of 1495 three-generational families, defined by IP siblings who have the same parents (mean sibship size is 1.67). SEDD IPs were followed over time from 1990, at an average age of 52 years, with a maximum mortality and morbidity follow-up of 25 years (1990-2015). In 2015, 694 (28%) IPs were deceased whereas 1,803 (72%) were still alive. Moreover, 1,190 (48%) IPs had a disease diagnosis whereas 1,307 (52%) IPs did not have a disease diagnosis (Figure 1B and Table 1B). From here we will refer to disease diagnoses as diseases, disease prevalence in cross-sectional analyses, and disease incidence in longitudinal analyses.

Recruitment

The Leiden Longevity Study (LLS) was initiated in 2002 to study the mechanisms that lead to exceptional survival. The LLS currently consist of 650 three-generational families, defined by siblings who have the same parents (Figure 1). Inclusion took place between 2002 and 2006 and initially started with the recruitment of living sibling pairs. Within a sibling pair, males were invited to participate if they were 89 years or older and females if they were 91 years or older. Inclusion was subsequently extended to the children of the sibling pairs and their partners. This study focuses on the children of the sibling pairs and their partners, referring to them as LLS IPs and partners. From their perspective, IPs were included if they had at least one long-lived parent and aunt or uncle (females ≥ 91 years and males ≥ 89 years). In total, 1,674 Index Persons (IPs, F3), 745 partners (F3), 1,295 parents (F2), 2,370 aunts and uncles (F2), 760 grandparents (F1), and 1,237 parents of the partners (F2) were included in this study. The LLS currently consist of 649 three-generational families, defined by IP siblings who have the same parents (Figure 1).

The Scanian Economic-Demographic Database (SEDD) is a longitudinal database covering five rural Scanian parishes and the city of Landskrona. It spans the period 1812-1967, with full coverage of the villages from 1812 and for Landskrona from 1904. The SEDD database was constructed using register-type data from catechetical examination registers and was updated with information on births, marriages, and deaths from church books. Unique person numbers were introduced in Sweden by 1947. Through these person numbers individuals can be followed in the national Swedish registration, introduced in 19671968. Persons who out-migrated from the research region before the introduction of the person number were linked to the 1950 Census and the Swedish Death Index. The obtained person numbers were subsequently used to track individuals in the Swedish national register for the period 1968-2015. The link to the Swedish Death Index yielded ancestral death dates anywhere in Sweden even for individuals who out-migrated from the research region before the person number or nationwide register data were introduced. At present (2022), the SEDD database contains 920,159 unique individuals.

Index person (IP) identification for this study happened in subsequent steps (Supplementary table 5). First, from the entire SEDD data we identified all persons (from here: IPs) who were part of the national register data in the years 1990-1995 and between ages 45-60, and followed them in the national registers for the period 1990-2015. Second, IPs were selected to have known grandparents on at least one side of the family (maternal or paternal), and whose parents were from an extinct birth cohort (born before 1915) to ensure complete information about their date of death. Third, we included lifespan information of their parents, aunts and uncles, and their grandparents. Fourth, IPs who were found in the hospital records in the year preceding their eligibility for the study (1989-1994) were excluded to minimize the number of IPs with existing conditions receiving hospital treatments. Lastly, partners of IPs were excluded to ensure mutually exclusive ancestral information. In

	total, 1,493 Index Persons (IPs, F3), 2,969 parents (F2), 5,830 aunts and uncles (F2), and 3,028 grandparents (F1) were included in this study. The SEDD consist of 1495 three-generational families, defined by IP siblings who have the same parents (Figure 1).
Ethics oversight	Leiden Longevity Study: In accordance with the Declaration of Helsinki, we obtained informed consent from all participants prior to their entering the study. Good clinical practice guidelines were maintained. The study protocol was approved by the ethical committee of the Leiden University Medical Center before the start of the study (P01.113).<br><br>SEDD: The SEDD has approval for research from Regionala etikprövningsnämnden, Lund, (dnr 161/2006, dnr 627/2010), and instructions from Datainspektionen, Stockholm (dnr 1999-2005).

Note that full information on the approval of the study protocol must also be provided in the manuscript.

Field-specific reporting

Please select the one below that is the best fit for your research. If you are not sure, read the appropriate sections before making your selection.

☒ Life sciences ☐ Behavioural & social sciences ☐ Ecological, evolutionary & environmental sciences

For a reference copy of the document with all sections, see nature.com/documents/nr-reporting-summary-flat.pdf

Life sciences study design

All studies must disclose on these points even when the disclosure is negative.

Sample size	We study 2,143 three-generational families (F1-F3) containing Index Persons (IPs; F3) their parents, aunts/uncles, grandparents, and partners, comprising 17,539 persons in total. This is currently the largest number of available individuals for intergenerational morbidity research.
Data exclusions	LLS: no persons were excluded from the data, though sub-selections were made for various analyses which are described in the metthods and results section.<br><br>SEDD: Index person (IP) identification for this study happened in subsequent steps (Supplementary table 5). First, from the entire SEDD data we identified all persons (from here: IPs) who were part of the national register data in the years 1990-1995 and between ages 45-60, and followed them in the national registers for the period 1990-2015. Second, IPs were selected to have known grandparents on at least one side of the family (maternal or paternal), and whose parents were from an extinct birth cohort (born before 1915) to ensure complete information about their date of death. Third, we included lifespan information of their parents, aunts and uncles, and their grandparents. Fourth, IPs who were found in the hospital records in the year preceding their eligibility for the study (1989-1994) were excluded to minimize the number of IPs with existing conditions receiving hospital treatments. Lastly, partners of IPs were excluded to ensure mutually exclusive ancestral information. In total, 1,493 Index Persons (IPs, F3), 2,969 parents (F2), 5,830 aunts and uncles (F2), and 3,028 grandparents (F1) were included in this study. The SEDD consist of 1495 three-generational families, defined by IP siblings who have the same parents (Figure 1).
Replication	All analyses were first conducted in the Leiden Longevity study. In a next step all disease incidence analyses involving the LRC score were repeated in the Swedish Register data. Our results were highly consistent between the two study populations, leading to the same conclusions. Replication of the LLS results in the Swedish data ensured:<br><br>1. that our results are robust for selection or (healthy) participant bias and other inclusion criteria related selections in the LLS, as the Swedish data does not apply any inclusion criteria and comes from the national registers which cover the entire country.<br>2. replication in independent data significantly reduces the risk of chance findings.<br>3. that our findings are robust for missing ancestral mortality data, as the Swedish data is based on (National) registers there is no missing mortality information,<br>4. together, the LLS and Swedish data cover the largest group of persons in the world to investigate disease incidence, with over 25 years of disease follow-up.
Randomization	Leiden Longevity Study<br>The Leiden Longevity Study (LLS) was initiated in 2002 to study the mechanisms that lead to exceptional survival. Inclusion took place between 2002 and 2006 and initially started with the recruitment of living sibling pairs. Within a sibling pair, males were invited to participate if they were 89 years or older and females if they were 91 years or older. Inclusion was subsequently extended to the children of the sibling pairs and their partners. In the current study, we focus on the children of the sibling pairs and their partners, referring to them as LLS IPs and partners. From their perspective, IPs were included if they had at least one long-lived parent and aunt or uncle (females ≥ 91 years and males ≥ 89 years). In total, 1,674 Index Persons (IPs, F3), 745 partners (F3), 1,295 parents (F2), 2,370 aunts and uncles (F2), 760 grandparents (F1), and 1,237 parents of the partners (F2) were included in this study. The LLS currently consist of 651 three-generational families, defined by IP siblings who have the same parents.<br><br>Scanian Economic-Demographic Database<br>The Scanian Economic-Demographic Database (SEDD) is a longitudinal database covering five rural Scanian parishes and the city of Landskrona. It spans the period 1812-1967, with full coverage of the villages from 1812 and for Landskrona from 1904. The SEDD database was constructed using register-type data from catechetical examination registers and was updated with information on births, marriages, and deaths from church books. Unique person numbers were introduced in Sweden by 1947. Through these person numbers individuals can be followed in the national Swedish registration, introduced in 1968. Persons who out-migrated from the research region before the introduction of the person number were linked to the 1950 Census and the Swedish Death Index. The obtained person numbers were subsequently used to track individuals in the Swedish national register for the period 1968-2015. The link to the Swedish Death Index yielded ancestral death

	dates anywhere in Sweden even for individuals who out-migrated from the research region before the person number or nationwide register data were introduced. At present (2022), the SEDD database contains 920,159 unique individuals.<br><br>Based on the proportion of long-lived ancestors, as measured using the Longevity Relatives Count (LRC) score, the following groups were identified: LRC_g1: IPs with an LRC ≥0.60, LRC_g2: IPs with an LRC [≥0.1 & <0.60], LRC_g3: partners with an LRC >0, and LRC_g4: partners with an LRC =0. All analyses are adjusted for age and sex as they are important confounders. It is needed to account for age to ensure that the association between delayed age-related disease onset and increasing number of long-lived ancestors, as measured with the LRC-score, is not due to age itself (for example because the Index persons with a higher proportion of long-lived ancestors are also younger). For this same reason, sex is included as a confounder. In the survival analyses, age is used as time scale and the potentially confounding effect of age is dealt with by accounting for left truncation.
Blinding	the LLS and SEDD are longitudinal cohort studies an the Swedish registry contains register data. As such, the study participants were not subject to an active intervention requiring blinding. Hence, study participants were not aware that they were identified as part of a specific group.

Reporting for specific materials, systems and methods

We require information from authors about some types of materials, experimental systems and methods used in many studies. Here, indicate whether each material, system or method listed is relevant to your study. If you are not sure if a list item applies to your research, read the appropriate section before selecting a response.

Materials & experimental systems

n/a	Involved in the study	
☒	☐	Antibodies
☒	☐	Eukaryotic cell lines
☒	☐	Palaeontology and archaeology
☒	☐	Animals and other organisms
☒	☐	Clinical data
☒	☐	Dual use research of concern

Methods

n/a	Involved in the study	
☒	☐	ChIP-seq
☒	☐	Flow cytometry
☒	☐	MRI-based neuroimaging
