# Supplementary material for: Increasing number of long-lived ancestors marks a decade of healthspan extension and healthier metabolomics profiles
Source: Nat Commun. 2023 Jul 27;14:4518. doi: 10.1038/s41467-023-40245-6 (PMC10374564; doi:10.1038/s41467-023-40245-6)
Supplement: Supplementary file 2 — Peer Review File [file 41467_2023_40245_MOESM2_ESM.pdf]

nature portfolio

Peer Review File

Increasing number of long-lived ancestors marks a decade of healthspan extension and healthier metabolomics profiles

Open Access This file is licensed under a Creative Commons Attribution 4.0 International License, which permits use, sharing, adaptation, distribution and reproduction in any medium or format, as long as you give appropriate credit to the original author(s) and the source, provide a link to the Creative Commons license, and indicate if changes were made. In the cases where the authors are anonymous, such as is the case for the reports of anonymous peer reviewers, author attribution should be to 'Anonymous Referee' followed by a clear attribution to the source work. The images or other third party material in this file are included in the article's Creative Commons license, unless indicated otherwise in a credit line to the material. If material is not included in the article's Creative Commons license and your intended use is not permitted by statutory regulation or exceeds the permitted use, you will need to obtain permission directly from the copyright holder. To view a copy of this license, visit http://creativecommons.org/licenses/by/4.0/.

Reviewer comments, first round

Reviewer #1 (Remarks to the Author):

This is a paper about the effects of familial longevity on disease incidence and time to disease. I have the following comments on this manuscript:

1. The 5th affiliation is not mentioned

2. Table 1A - percent of women in the last 2 columns looks small

3. It looks like many parents in LLS sample are missing (what is the percentage of missing parents?). Can it cause bias? (only 1295 parents for 1674 IPs). If there are many IPs in the same family, then what is the mean sibship size?

4. What specific diseases comprise the group of metabolic diseases?

5. What is the accuracy of LRC estimate when some portion of parents and other relatives is missing?

6. "capturing delayed mortality" - may be to say "lower mortality", because mortality risk exists at all ages (row 269).

7. It is not surprising that effects of long lived relatives on morbidity risk are stronger in SEDD data, because SEDD started to analyze risk at younger ages (7 years earlier than LLS and hence had almost twice as low risk of mortality at the outset). It was demonstrated that relative differences in mortality between people having and not having long-lived relatives decrease with age (see Protective Effects of Familial Longevity Decrease with Age and Become Negligible for Centenarians. Gerontology Series A-Biological Sciences & Medical Sciences. 2022). The same explanation may work for morbidity risk as well.

8. This manuscript needs some editing (see, for example, line 468)

Reviewer #2 (Remarks to the Author):

(1) In general, the description of the statistical methods is minimal and not clear. For example, a mixed model was used but it is unclear what are the fixed effects and random effects in the model. Sensitivity analysis was mentioned but no information on the specific type of sensitivity analysis.
(2) It is not clear whether multiple testing has been considered.
(3) It is unclear how the missing data was handlded, and whether clinical and demographic variables have been adjusted in the analyses.
(4) Since the LRC score is crucial in the manuscript, it would be helpful to describe how to calculate the score.
(5) How exactly has the validation been done?
(6) Any specific reason why the data, even the deidentified, cannot be publicly available?

Reviewer #3 (Remarks to the Author):

Through a detailed exploration on the influence of the number of long-lived ancestors on the offspring's disease burden in two three-generational data based on family and the whole population perspectives, respectively, the authors discovered some interesting findings. This is a complete story to reveal the longevity codes. However, several questions may need to be addressed:

A general issue is the data quality. Since a lot of info was attracted from existing databases including disease diagnosis, medication use, etc., which were the key outcomes used in this study, whether/how the missingness affected the analysis was unclear. For instance, SES might be one important confounding factor in terms of data quality (persons with high SES are more likely to have medical documentations and records). This is just an example.
Second, some nonsignificant results were shown in this study, although we know P value should not be a golden standard. Since multiple tests were also performed in this study, this issue became much more serious.

Some other specific issues.
1. Does the authors consider the influence of maternal or paternal heredity differences on the offspring's disease burdens? These differences may also influence the occurrence and the onset time of certain diseases. Besides, the disease history of the grandparents or parents of IPs may also need to be considered.
2. The authors performed quantitative analyses between LRC scores and the risk of disease burden, though this relationship has statistical significance; however, we wonder whether this relationship was in a linear trend.
3. We wonder whether the authors considered the different weights from different generation relatives when calculating the LRC scores.
4. The category of diseases should be clarified, age related disease includes metabolic disease.
5. In the LLS database, the partner of IPs was as the matched group; this is an advantage for this study because the partners shared a relatively similar social-economic environment with IPs. However, other different factors between the IPs and their partners should also be considered in the statistical models, such as occupation, and even the early life experiences, etc.
6. As similar to Question4, in the SEDD database, more influential factors should be considered for the analysis was not family-based (the participants shared a similar life environment).
7. We noticed that in the LLS database, the number of grandparents(F1) was only half of IPs, which is a contrast with that of the SEDD database, whether these missing ancestor's information would influence the accuracy of LRC scores, the authors need to consider.
8. In supplementary Tables 2 and 3, the units of the labels were (mean), while the results displayed in the tables were proportions; and thus these errors need to be corrected.

Reviewer 1

This is a paper about the effects of familial longevity on disease incidence and time to disease. I have the following comments on this manuscript:

Comment A

The 5th affiliation is not mentioned.

Response

We thank the reviewer for noticing that the fifth affiliation is not mentioned. By accident we denoted P. Eline Slagboom[1,5] instead of P. Eline Slagboom[1,4].

Implementations

We changed the affiliation indicator for P. Eline Slagboom into: P. Eline Slagboom[1,4]

Comment B

Table 1A - percent of women in the last 2 columns looks small.

Response

We thank the reviewer for identifying these editorial errors in Table 1. The percentage should have been 50% instead of 5%.

Implementations

We adjusted the percentage of 5% to the correct 50% and checked the other figures and tables, as well as the manuscript for editorial errors and subsequently adjusted any identified errors.

Comment C

It looks like many parents in LLS sample are missing (what is the percentage of missing parents?). Can it cause bias? (only 1295 parents for 1674 IPs. If there are many IPs in the same family, then what is the mean sibship size?

Response

We thank the reviewer for raising this point as it made us realize that we have to provide additional family information in Table 1 and the paper. The LLS data consists of 1674 IPs from 651 families. Indeed multiple IPs belonged to the same family (see Figure 1 which combines with Table 1). The average sibship size of the LLS IPs is 2.58. Only for 2 IPs no parents were identified (0.1%), which is too little to cause bias.

Implementations

We have updated Table 1, Figure 1, and the manuscript with additional family information so that it becomes more clear that we study multiple IPs descending from the same family in both the LLS and SEDD. We further mention the number of families, and the mean sibship size more clearly in the study population descriptions (line 109-129).

Comment D
What specific diseases comprise the group of metabolic diseases?

Response
We would like to refer the reviewer to Supplementary Table 4, which we mention in the third paragraph of the methods section, where the disease categories are specified and the corresponding ICD-10 codes are provided:

Supplementary Table 4: disease groups based on ICD scores for GP data

**Diseases (ICD10-code)**	**Age related**	**Metabolic**	**Malignancies**
TIA (I60-I69)	X	X	
CVA (I63)	X	X	
AP (I20)	X	X	
MI (I21)	X	X	
Hypertension (I10)	X	X	
Diabetes (E10–E14)	X	X	
Prostate (C61)	X		X
Mamma (C50)	X		X
Lung (C34)	X		X
Colon (C18-C20)	X		X
Other (C00-D48)	X		X

Implementations
We realized that we only referred to this Supplementary Table in the methods section. For clarity, we now refer to this Supplementary Table 4 also in the results and discussion section, when mentioning the age-related disease for the first time (line 143-144 and line 280).

Comment E
What is the accuracy of LRC estimate when some portion of parents and other relatives is missing?

Response
The LRC score is based on multiple generations, covering parents, aunts and uncles, and grandparents, which provides protection against the presence of missing data and makes the score very robust for missing data.

Missing ancestral data may arise mainly because of unidentified ancestors, or missing mortality information. To understand the impact of missing ancestral data, we conducted an illustrative simulation study where we compared the LRC score precision in a large family situation (average ancestor size of 7.5) compared to a small family situation (average ancestor size of 4). This situation mimics the effect of missing ancestral mortality information; the family size is reduced. The prediction precision (defined as the accuracy to detect truly long-lived families; True Positive Rate (TPR)) was very similar, with only a 4% lower accuracy in the small family situation. To make this more concrete: consider a person with 8 ancestors of which only 4 ancestors have available mortality information. In this case there is a 4% lower accuracy in LRC-score compared to when all 8 ancestors had available mortality information. In addition, missing data has almost no impact in increasing false positive detection (False Positive Rate (FPR)) of long-lived families. We added the R-script of the simulation study for the reviewers.

To further rule out any interference of available ancestral data, our LLS findings were replicated in the Swedish register data (SEDD data) where all the Swedish index persons had available parents, aunts/uncles and grandparent (no missing data). The fact that our conclusions remain identical indicates good performance of the LLS approach.

Implementations

In the discussion methods section we have highlighted the importance of multiple ancestral generations and replication with the Swedish register data more clearly (line 346-349and line line 616-632).

Comment F

"capturing delayed mortality" - may be to say "lower mortality", because mortality risk exists at all ages (row 269).

Response

We agree with the reviewer that it would be more appropriate to rephrase "delayed mortality" as the statement is indeed about mortality risk

Implementations

We rephrased the sentence into: "We conclude that an increasing number of long-lived ancestors, as measured with the LRC score, is a quantitative indicator of familial longevity, capturing decreased mortality risk, protection against multimorbidity, and improved health in selected families as well as the population." (line 287-290).

Comment G

It is not surprising that effects of long lived relatives on morbidity risk are stronger in SEDD data, because SEDD started to analyze risk at younger ages (7 years earlier than LLS and hence had almost twice as low risk of mortality at the outset). It was demonstrated that relative differences in mortality between people having and not having long-lived relatives decrease with age (see Protective Effects of Familial Longevity Decrease with Age and Become Negligible for Centenarians. Gerontology Series A-Biological Sciences & Medical Sciences. 2022. The same explanation may work for morbidity risk as well.

Response

We agree with the reviewer that the younger average follow-up age, as well as the longer follow-up period in SEDD is likely one of the mechanisms explaining the slightly stronger effects, though please note that the conclusions are identical for the different datasets (SEDD and LLS). It is, however, not necessarily the most obvious explanation as we investigate age-related diseases which generally onset during the later-half of mid-life[3]. Hence, we discuss possible explanations for the differences in effect size between the LLS and SEDD in detail in the third and fourth paragraph of the discussion. We explain that the difference in outcome; GP diagnosis in LLS compared to hospitalization in SEDD as well as the comparison with index persons and partners in the LLS can lead to more conservative (smaller) effect sizes in the LLS than in the SEDD. These explanations are, in line with the work of Christensen et al. (2017) showing that comparisons with partners leads to more conservative estimates than comparisons with the general population[4].

Implementations

We incorporated a sentence referring to this possible explanation and the reference in the discussion section where we discuss the differences in effect size between LLS and SEDD (line 318-325).

Comment H

This manuscript needs some editing (see, for example, line 468).

Response

We agree with the reviewer that some errors slipped through our editorial checks

Implementations

We have reviewed the entire paper, figures, and tables in order to limit the number of editorial mistakes. in particular we would like to mention that we have removed paragraph 3 from the results section because this information was redundant in hindsight. We refer to the revised manuscript for all adjustments.

Reviewer 2

Comment A

In general, the description of the statistical methods is minimal and not clear. For example, a mixed model was used but it is unclear what are the fixed effects and random effects in the model. Sensitivity analysis was mentioned but no information on the specific type of sensitivity analysis.

Response

We agree with the reviewer that the statistical methods section may benefit from some clarification, though we do described in line 497-499 of the initial manuscripts that we consider the F3 index persons with the same parents as a family and that the family indicator is modeled as a random effect/frailty in all the statistical models to adjust for within-family relations of the F3 IPs. However, the notation was unclear at some points so we have clarified the results and entire statistical section.

Implementations

We have clarified the results section, updated the entire statistical methods section, and added descriptions of the specific variables in the models.

Comment B

It is not clear whether multiple testing has been considered.

Response

We presume the reviewer would like to know whether it was necessary to correct for multiple testing in order to avoid false positive results in our study. Identifying false positive results can be a serious issue when investigating a large number of independent variables one by one, which for example happens in Genome Wide Association (GWA) studies or Metabolome Wide Association studies. In these studies, repeated testing is used to select the most promising SNPs or metabolites given the specific outcome under investigation. In other words, an explicit distinction between interesting and uninteresting findings is made in these settings. An important danger to such an approach is the increasing (with the number of tests) chance of finding spurious significant associations. For example, when testing the association between 20 SNPs and a phenotype using a p-value threshold of 0.05, the probability of calling 1 or more false positives by chance is 64%. Note that this calculation assumes independent tests, the type I error inflation is <u>much lower</u> if tests are correlated.

The way our analyses are conducted and interpreted is quite different from the GWA and other similar studies as discussed above. We are not in a setting where a large number of different hypotheses are tested and the most interesting findings are highlighted. We have a single hypothesis under investigation: the existence of an association between family history of longevity and delayed disease incidence. To investigate this hypothesis, we use a number of similar outcomes (See Table below for a detailed summary), an approach that safeguards against false positive results. To illustrate: we show that metabolic diseases are (1) delayed at time point 1 (onset of first metabolic disease), (2) time point 2 (onset of second metabolic disease), and (3) between time point 1 and 2 (onset of second disease after diagnosis of a first disease). In addition, (4) the odds to use metabolic disease related medication (ATC-C

code medication) is reduced for those with a family history of longevity. Finally, (5) we find the same result when replicating the analyses in independent data (the Swedish Register data; SEDD), which is the best way to avoid false positive findings. In total 5 out of the 5 ways confirm the association between familial longevity and delayed metabolic disease incidence.

As we are not highlighting some results over others and all outcomes used to test our hypothesis provide similar results, our analyses are unlikely to be exposed to multiple testing problems. As mentioned above, multiple testing related false positive findings would have been an issue if results would differ from some outcomes to others, and we would highlight only some of such significant results. As this is not what we do, explicit correction for multiple testing (e.g. providing corrected p-values) does not seem an appropriate measure in our study.

Special mention deserves the case of malignancies for which we have not found a clear tendency, and results differ depending on the chosen outcome. We believe that our conclusions regarding malignant diseases are very carefully phrased in the discussion section, paragraph 4.

**Longevity definitions**	**Outcomes Medication use**	**Outcomes diseases T1**	**Outcomes diseases T2**	**Outcomes diseases T1-T2**	**Metabohealth**
**LLS**					
Part/offspring	1. ATC-A<br>2. ATC-B<br>3.ATC-C	1. age-related diseases (combination of metabolic and malignant diseases)<br>2. metabolic diseases<br>3. malignant diseases	1. age-related diseases (combination of metabolic and malignant diseases)<br>2. metabolic diseases<br>3. malignant diseases	1. age-related diseases (combination of metabolic and malignant diseases)<br>2. metabolic diseases<br>3. malignant diseases	
LRC part/offspring	1. ATC-A<br>2. ATC-B<br>3.ATC-C	1. age-related diseases (combination of metabolic and malignant diseases)<br>2. metabolic diseases<br>3. malignant diseases	1. age-related diseases (combination of metabolic and malignant diseases)		1. metabo-health score
LRC continuous		1. age-related diseases (combination of metabolic and malignant diseases)<br>2. metabolic diseases<br>3. malignant diseases			
**SEDD**					
LRC continuous		1. age-related diseases (combination of metabolic and malignant diseases)<br>2. metabolic diseases<br>3. malignant diseases			

Implementations

We have updated the discussion and methods section, underlining the strength of our study design, both in terms of approach and replication in independent data, to avoid false positive results (line 346-349 and line 616-632).

Comment C

It is unclear how the missing data was handled, and whether clinical and demographic variables have been adjusted in the analyses.

Response

This question contains two components. One about how missing data was handled and one about the analyses

Missing data

Missing data can occur on two levels, (1) on the level of the Index Persons and (2) on the level of the ancestors of the index persons. This comment partly overlaps with Comment C of reviewer 1.

Missing data on the level of the index persons is described in Table 1 (including the legend), the methods section and throughout the results. Note that in the light of Comment C of reviewer 1, we added the number sibships to Table 1, because we recognized that without that information one might think that there is parental data missing. For all our analyses, persons with relevant missing data were removed from the analyses. For example, from the 2419 IPs in the LLS, 683 had missing disease incidence data and were subsequently removed from the analyses. For the Swedish data (SEDD) there was no missing data as the data comes from the National Swedish Registers covering the entire population. Table 1 provides the total number of persons in the LLS and SEDD data and the results section of the manuscript as well as the Tables and Figures describe the exact number of persons included in a specific analysis.

Censored and missing data on the level of the ancestors is described in the lifetable paragraph of the methods section. If ancestors of IPs had right-censored mortality data, meaning that an age of last observation was available but no age at death, we used single imputation based on the Dutch lifetables provided by statistics Netherlands to estimate age at death. Ancestors without any mortality data at all were removed from calculating the Longevity Relatives Count Score, so it implies a reduction in the family size available to compute the LRC-score. Table 1 further shows that for the SEDD data there was no missing mortality information on the level of the ancestors.

To understand the impact of missing ancestral data (for example because of unidentified ancestors, or missing mortality information), we conducted an illustrative simulation study where we compared the LRC score precision in a large family situation (average ancestor size of 7.5) compared to a small family situation (average ancestor size of 4). This situation mimics the effect of missing ancestral mortality information; the family size is reduced. The prediction precision (defined as the accuracy to detect truly long-lived families; True Positive Rate (TPR)) was very similar, with only a 4% lower accuracy in the small family situation. To make this more concrete: consider a person with 8 ancestors of which only 4 ancestors have available mortality information. In this case there is a 4% lower accuracy in LRC-score compared to when all 8 ancestors had available mortality information. In addition, missing data has almost no impact in increasing false positive detection (False Positive Rate (FPR)) of long-lived families. We added the R-script of the simulation study for the reviewers. To further rule out any interference of available ancestral data, our LLS findings were replicated in the Swedish register data (SEDD data) where all the Swedish index persons had available

parents, aunts/uncles and grandparent (no missing data). The fact that our conclusions remain identical indicates good performance of the LLS approach.

Adjustment of analyses

Whether analyses have been adjusted for clinical and demographic variables is an important question which we address in the statistics paragraph of the methods section.

All analyses are adjusted for age and sex as they are important confounders. It is needed to account for age to ensure that the association between delayed age-related disease onset and increasing number of long-lived ancestors, as measured with the LRC-score, is not due to age itself (for example because the Index persons with a higher proportion of long-lived ancestors are also younger). For this same reason, sex is included as a confounder. In the survival analyses, age is used as time scale and the potentially confounding effect of age is dealt with by accounting for left truncation. All analyses on disease incidence are unadjusted for medication use as medication use typically follows after disease diagnosis and because disease onset and the onset of medication use are highly correlated and likely to measure the same construct. The analysis of the association between the MetaboHealth score and the LRC-based partner/offspring groups is adjusted for medication use as medication use, besides age and sex, may directly impact the circulating metabolite levels.

Our main purpose was to show that an increasing number of long-lived ancestors associates with a delayed disease incidence, multimorbidity, medication use, and healthy metabolomics profile. The purpose of this study was not to identify the mechanisms underlying the observed delayed disease incidence. Demographic, genomic, social, behavioral factors, and their complex interactions can all be expected to (partly) explain the observed delayed disease incidence and healthy metabolomics profile, and will be the focus of future papers.

Implementations

We have more clearly described data availability in the legend of Figure 1 and Table 1 as well as the methods section. In addition, we updated the entire statistics section to clarify our analyses, including the description of all variables included in the models.

Comment D

Since the LRC score is crucial in the manuscript, it would be helpful to describe how to calculate the score.

Response

We agree with the reviewer that our method to summarize an increasing proportion of long-lived ancestors, the LRC score, should be explained in the paper instead of only referring to previous work that describes the LRC score. We will add a description of how to calculate the LRC-score to this paper.

Implementations

We added a more detailed description and the formula to calculate the LRC score in the methods section (line 502-522).

Comment E

How exactly has the validation been done?

Response

We thank the reviewer for asking clarification about validating the results from the Leiden Longevity Study in the Swedish Register data. All analyses were first conducted in the Leiden Longevity study. In a next step all disease incidence analyses involving the LRC score were repeated in the Swedish Register data. Our results were highly consistent between the two study populations, leading to the same conclusions. Replication of the LLS results in the Swedish data ensured:

1. that our results are robust for selection or (healthy) participant bias and other inclusion criteria related selections in the LLS, as the Swedish data does not apply any inclusion criteria and comes from the national registers which cover the entire country.
2. replication in independent data significantly reduces the risk of chance findings.
3. that our findings are robust for missing ancestral mortality data, as there is no missing mortality information in the SEDD.
4. together, the LLS and Swedish data cover the largest group of persons in the world to investigate disease incidence, with over 25 years of disease follow-up.

The comment, however, made us realize that the term validation can cause confusion because validation is often used to show that an instrument/tool developed in a certain training set, accurately measures what it should measure in an independent validation set. In our case it is about observing the same effects in the LLS and the Swedish data, making replication a more appropriate term.

Implementations

We changed the word “validation” into “replication” throughout the paper and added a more detailed description of replication in the Swedish data to the methods section (line 616-632). We further added a discussion of the benefits of replication to the discussion section of the paper.

Comment F

Any specific reason why the data, even the deidentified, cannot be publicly available?

Response

Both databases are available for research purposes upon reasonable request.

LLS

Based on European and Dutch privacy legislation as well as the Informed Consent signed by the study participants the data are not allowed to be stored online. Hence, researchers can request data access and analyze the data, in collaboration with the LLS research group, on a secure server in the Leiden University Medical Center, the Netherlands.

SEDD/Swedish Register data

The Swedish data consists of two parts, (1) the historical part containing family trees and (2) the part of the national Swedish Register data. Both parts are connected to each other.

Similar to the LLS, European and Swedish privacy legislation prohibits public availability of the historical (part 1) data. However, access to the data can be provided upon request. For the Swedish National Register data privacy legislation is even more strict than normal privacy legislation to make sure that personal data is protected; a set of specific laws regulates access to the Swedish National Register data and requirements involve among others ethical permission for the researcher as well as an affiliation to a Swedish research institute.

Reviewer 3

Through a detailed exploration on the influence of the number of long-lived ancestors on the offspring's disease burden in two three-generational data based on family and the whole population perspectives, respectively, the authors discovered some interesting findings. This is a complete story to reveal the longevity codes. However, several questions may need to be addressed:

Comment A

A general issue is the data quality. Since a lot of info was attracted from existing databases including disease diagnosis, medication use, etc., which were the key outcomes used in this study, whether/how the missingness affected the analysis was unclear. For instance, SES might be one important confounding factor in terms of data quality (persons with high SES are more likely to have medical documentations and records). This is just an example.

Response

We presume the reviewer would like to know whether it is possible that specific groups in the data (for example persons with a low SES) more often have missing data than other persons (for example persons with a high SES).

It is very unlikely in the Netherlands that persons with a low SES are less likely to have medical documentation/records. Health insurance is obligatory and subsidized for lower SES people. Moreover, when a disease occurs persons go for free (no costs involved) to the general practitioner (GP) for diagnosis and appropriate reference to a medical specialist if necessary. We retrieved the Dutch disease data directly from the GPs, making it highly unlikely that there is a differential missing data pattern for low SES persons than high SES persons.

We further ruled out effects of missing data by replicating our LLS findings in the National Swedish Register data (SEDD data), which cover the entire population, using the hospitalization records. This approach is unique because the register data are not affected by missing data or selection/participation bias for that matter. The combination of LLS (GP records) and SEDD (hospitalization records) make our study design unique in terms of data quality and availability which is therefore one of the strengths of our study.

Implementations

We have more clearly described data availability in the legend of Figure 1 and Table 1 as well as the methods section (line 610-629). In addition, we updated the entire statistics section to clarify our analyses, including the description of all variables included in the models. We further added a more detailed description of replication in the Swedish data to the methods section and added a discussion of the benefits of replication to the discussion section of the paper (line 616-632 and line 346-349).

Comment B

Second, some nonsignificant results were shown in this study, although we know P value should not be a golden standard. Since multiple tests were also performed in this study, this issue became much more serious.

Response

We presume the reviewer would like to know whether it was necessary to correct for multiple testing in order to avoid false positive results in our study. Identifying false positive results can be a serious issue when investigating a large number of independent variables one by one, which for example happens in Genome Wide Association (GWA) studies or Metabolome Wide Association studies. In these studies, repeated testing is used to select the most promising SNPs or metabolites given the specific outcome under investigation. In other words, an explicit distinction between interesting and uninteresting findings is made in these settings. An important danger to such an approach is the increasing (with the number of tests) chance of finding spurious significant associations. For example, when testing the association between 20 SNPs and a phenotype using a p-value threshold of 0.05, the probability of calling 1 or more false positives by chance is 64%. Note that this calculation assumes independent tests, the type I error inflation is <u>much lower</u> if tests are correlated.

The way our analyses are conducted and interpreted is quite different from the GWA and other similar studies as discussed above. We are not in a setting where a large number of different hypotheses are tested and the most interesting findings are highlighted. We have a single hypothesis under investigation: the existence of an association between family history of longevity and delayed disease incidence. To investigate this hypothesis, we use a number of similar outcomes (See Table below for a detailed summary), an approach that safeguards against false positive results. To illustrate: we show that metabolic diseases are (1) delayed at time point 1 (onset of first metabolic disease), (2) time point 2 (onset of second metabolic disease), and (3) between time point 1 and 2 (onset of second disease after diagnosis of a first disease). In addition, (4) the odds to use metabolic disease related medication (ATC-C code medication) is reduced for those with a family history of longevity. Finally, (5) we find the same result when replicating the analyses in independent data (the Swedish Register data; SEDD), which is the best way to avoid false positive findings. In total 5 out of the 5 ways confirm the association between familial longevity and delayed metabolic disease incidence.

As we are not highlighting some results over others and all outcomes used to test our hypothesis provide similar results, our analyses are unlikely to be exposed to multiple testing problems. As mentioned above, multiple testing related false positive findings would have been an issue if results would differ from some outcomes to others, and we would highlight only some of such significant results. As this is not what we do, explicit correction for multiple testing (e.g. providing corrected p-values) does not seem an appropriate measure in our study.

Special mention deserves the case of malignancies for which we have not found a clear tendency, and results differ depending on the chosen outcome. We believe that our conclusions regarding malignant diseases are very carefully phrased in the discussion section, paragraph 4.

**Longevity definitions**	**Outcomes Medication use**	**Outcomes diseases T1**	**Outcomes diseases T2**	**Outcomes diseases T1-T2**	**Metabohealth**
**LLS**					
Part/offspring	1. ATC-A<br>2. ATC-B<br>3.ATC-C	1. age-related diseases (combination of metabolic and	1. age-related diseases (combination of metabolic and	1. age-related diseases (combination of metabolic and	

		malignant diseases)<br>2. metabolic diseases<br>3. malignant diseases	malignant diseases)<br>2. metabolic diseases<br>3. malignant diseases	malignant diseases)<br>2. metabolic diseases<br>3. malignant diseases	
LRC part/offspring	1. ATC-A<br>2. ATC-B<br>3.ATC-C	1. age-related diseases (combination of metabolic and malignant diseases)<br>2. metabolic diseases<br>3. malignant diseases	1. age-related diseases (combination of metabolic and malignant diseases)		1. metabo-health score
LRC continuous		1. age-related diseases (combination of metabolic and malignant diseases)<br>2. metabolic diseases<br>3. malignant diseases			
**SEDD**					
LRC continuous		1. age-related diseases (combination of metabolic and malignant diseases)<br>2. metabolic diseases<br>3. malignant diseases			

Implementations

We have updated the discussion and methods section, underlining the strength of our study design, both in terms of approach and replication in independent data, to avoid false positive results (line 346-349 and line 616-632).

Comment C

Does the authors consider the influence of maternal or paternal heredity differences on the offspring's disease burdens? These differences may also influence the occurrence and the onset time of certain diseases. Besides, the disease history of the grandparents or parents of IPs may also need to be considered.

Response

As described in the lifetable paragraph of the methods section we indeed take maternal and paternal differences in longevity into account by using birth cohort and sex based survival percentiles to define longevity. For example, a male born in the year 1900 belongs to the top 10% survivors of his birth cohort if he reaches an age of 86 years. A female born in 1900 belongs to the top 10% survivors if she reaches an age of 90 years. In a series of publications we showed that, using this approach we showed that the heritability/intergenerational transmission of longevity does not differ between males and females[1,2,5].

It is not entirely clear why considering disease history of parents or grandparents would benefit our study. Moreover, we do not have multiple generations of disease data available. There is a vast literature showing that long-lived individuals have less diseases and compressed morbidity towards the end of life than population controls[6]. In other words, survival to exceptional ages and delayed disease incidence seem to be highly interrelated. We capitalize on these findings to for the first time show that by taking ancestral mortality of study participants into account there is a very strong association between the increasing number of long-lived ancestors and an increasingly delayed disease incidence of the study participants. Hence, we are the first to show a quantitative linear association between ancestral longevity and delayed disease incidence of study participants.

Implementations

We added an explaining sentence to the lifetable paragraph of the methods section in the paper (line 493).

Comment D

The authors performed quantitative analyses between LRC scores and the risk of disease burden, though this relationship has statistical significance; however, we wonder whether this relationship was in a linear trend.

Response

We indeed tested for linearity using the ggcoxfunctional() function of the survminer package in R using a regular Cox model without family specific random effects. The function ggcoxfunctional() provides a graph of the continuous covariate under investigation (theLRC-score in our case) against the martingale residuals of null Cox model. If the effect is linear you expect a roughly linear line between lying in between the residual points. Below, we show the Martingale residuals graph obtained for the analysis of time to first age-related disease.

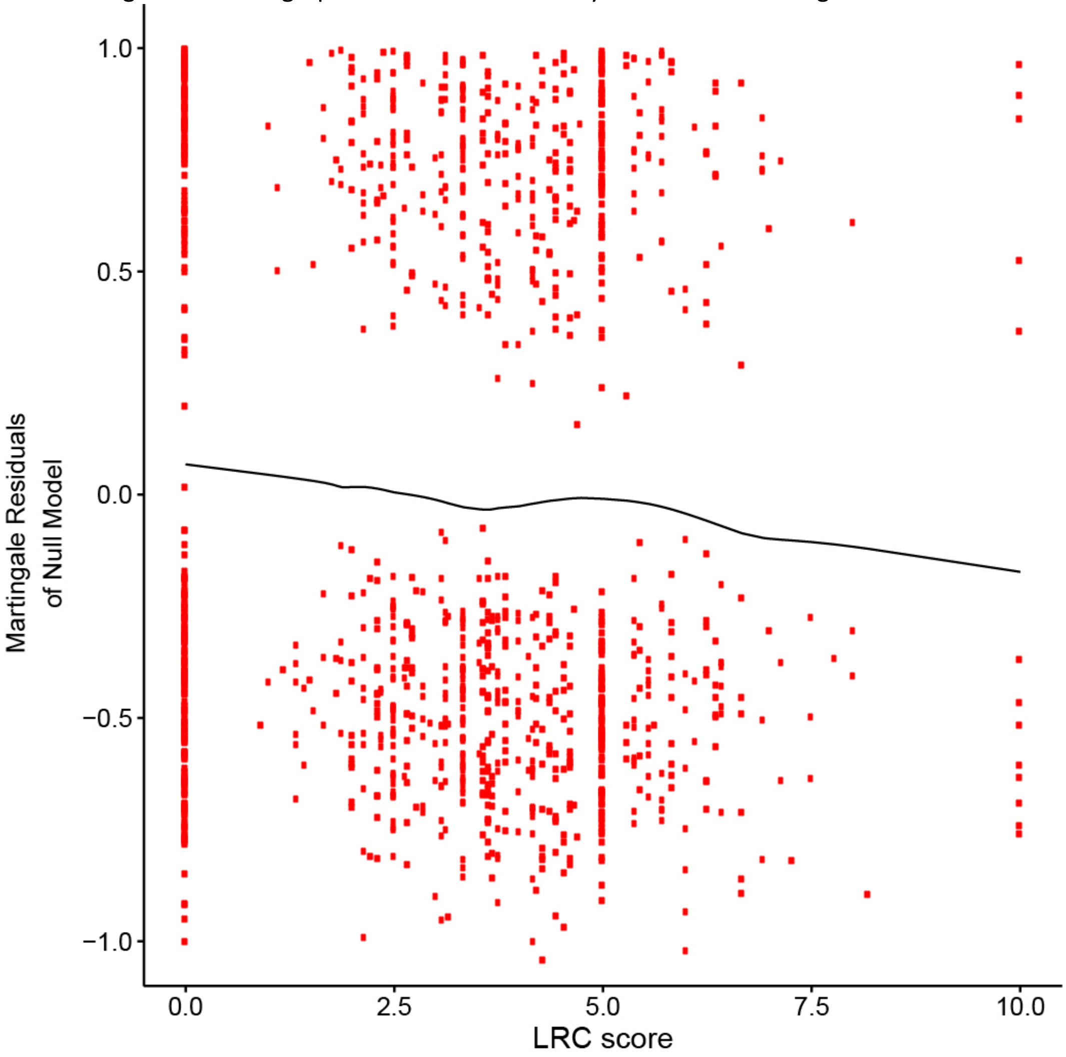

It can be seen that there is a nice linear association between age-related diseases and an increasing number of long-lived ancestors, as measured by the LRC score. A same linear trend was observed for metabolic and malignant diseases, although the line is much more horizontal for malignant diseases, reflecting the very small effect of malignancies. In addition, Figure 3 panel B and Table 3 clearly show that groups with an increasing number of long-lived ancestors (group 2 has a higher LRC score than group 1 and group 2 has a higher LRC score than group 2, etc.) have an increasing delay in first disease and multimorbidity.

Implementations

We added clarification of checking the linearity assumption to the statistics section (line 634-640).

Comment E

We wonder whether the authors considered the different weights from different generation relatives when calculating the LRC scores.

Response

The LRC score is indeed weighted by the genetic distance between study participants and ancestors. For example parents count for 50% and grandparents for 25% and so on, the more distantly related the ancestors are. We described this two times throughout the paper. In paragraph 8 of the results: "Increasing numbers of long-lived ancestors indicate a later disease onset in LLS and SEDD" and paragraph 9 of the methods: "Scores".

Implementations

We now more clearly describe the construction and interpretation of the LRC score by incorporating the formula and it's description in the methods section (line 502-522).

Comment F

The category of diseases should be clarified, age related disease includes metabolic disease.

Response

We would like to refer the reviewer to Supplementary Table 4 where the disease categories are specified and the corresponding ICD-10 codes are provided:

Supplementary Table 4: disease groups based on ICD scores for GP data

**Diseases (ICD10-code)**	**Age related**	**Metabolic**	**Malignancies**
TIA (I60-I69)	X	X	
CVA (I63)	X	X	
AP (I20)	X	X	
MI (I21)	X	X	
Hypertension (I10)	X	X	
Diabetes (E10–E14)	X	X	
Prostate (C61)	X		X
Mamma (C50)	X		X
Lung (C34)	X		X
Colon (C18-C20)	X		X
Other (C00-D48)	X		X

The third paragraph of the methods section further describes that: “Diseases were clustered into 3 groups based on the International Statistical Classification of Diseases and Related Health Problems (ICD-10) codes, (1) metabolic diseases, (2) malignant diseases, and (3) age related diseases, which are the combination of metabolic and malignant diseases.”. To increase the number of observations and therefore statistical power and because our focus is on age-related diseases (all diseases have a known association with aging) the combined age-related disease category was constructed.

Implementations

We realized that we only referred to this Supplementary Table in the methods section. To accommodate the reviewer we refer to this Supplementary Table 4 also in the results and discussion section, when mentioning the age-related disease for the first time (line 143-144 and line 280).

Comment G

In the LLS database, the partner of IPs was as the matched group; this is an advantage for this study because the partners shared a relatively similar social-economic environment with IPs. However, other different factors between the IPs and their partners should also be considered in the statistical models, such as occupation, and even the early life experiences, etc.
As similar to Question4, in the SEDD database, more influential factors should be considered for the analysis was not family-based (the participants shared a similar life environment).

Response

This is an important question which we address in the statistics paragraph of the methods section.

All analyses are adjusted for age and sex as they are important confounders. It is needed to account for age to ensure that the association between delayed age-related disease onset and increasing number of long-lived ancestors, as measured with the LRC-score, is not due to age itself (for example because the Index persons with a higher proportion of long-lived ancestors are also younger). For this same reason, sex is included as a confounder. In the survival analyses, age is used as time scale and the potentially confounding effect of age is dealt with by accounting for left truncation. All analyses on disease incidence are unadjusted for medication use as medication use typically follows after disease diagnosis and because disease onset and the onset of medication use are highly correlated and likely to measure the same construct. The analysis of the association between the MetaboHealth score and the LRC-based partner/offspring groups is adjusted for medication use as medication use may directly impact the metabolite levels.

Our main purpose was to show that an increasing number of long-lived ancestors associates with a delayed disease incidence, multimorbidity, medication use, and healthy metabolomics profile. The purpose of this study was not to identify the mechanisms underlying the observed delayed disease incidence. Demographic, genomic, social, behavioral factors, and their complex interactions can all be expected to (partly) explain the observed delayed disease incidence and healthy metabolomics profile, and will be the focus of future papers. Adding additional covariates such as socio-economic status or including genomic data can help to answer the question why an increasing number of long-lived ancestors associates with an increasingly delayed disease incidence. Though, answering this question would

require a more complex design to capture gene environment interactions than adding covariates to our models.

In the discussion we do provide a direction for future research. We describe that the delayed disease incidence observed in members of long-lived families is likely due to genetic and socio-behavioral factors, as well as their interaction. For this reason it is interesting to compare the effect sizes of the Leiden Longevity Study, where index persons were compared to their partners, with the effect sizes of the Swedish Register data, where this was not the case. As we outline in the discussion, such a comparison may give a broad indication to what extent genetic and socio-behavioral factors may drive the association between an increasing number of long-lived ancestors and an increased delay in disease onset of the index persons.

Comment H

We noticed that in the LLS database, the number of grandparents(F1) was only half of IPs, which is a contrast with that of the SEDD database, whether these missing ancestor's information would influence the accuracy of LRC scores, the authors need to consider.

Response

The reviewer addresses an interesting point. In the SEDD data there are indeed slightly more grandparents identified than in the LLS. This has 2 reasons: (1) there are more families in SEDD than in LLS, as shown by the smaller sibship sizes and (2) because in SEDD we could go to the grandparent generation via the maternal and paternal line. The LRC score was originally constructed and validated on identical ancestor availability as the LLS data[2] so it is unlikely to be inaccurate in the LLS, but accuracy can increase with the availability of additional ancestors. This principle is similar to the size of a database/sample where larger samples will provide more precise estimates, reflected in smaller confidence intervals.

To better understand the impact of missing ancestral data (for example because of unidentified ancestors, or missing mortality information), we conducted an illustrative simulation study where we compared the LRC score precision in a large family situation (average ancestor size of 7.5) compared to a small family situation (average ancestor size of 4). This situation mimics the effect of missing ancestral mortality information; the family size is reduced. The prediction precision (defined as the accuracy to detect truly long-lived families; True Positive Rate (TPR)) was very similar, with only a 4% lower accuracy in the small family situation. To make this more concrete: consider a person with 8 ancestors of which only 4 ancestors have available mortality information. In this case there is a 4% lower accuracy in LRC-score compared to when all 8 ancestors had available mortality information. In addition, missing data has almost no impact in increasing false positive detection (False Positive Rate (FPR)) of long-lived families. We added the R-script of the simulation study for the reviewers. To further rule out any interference of available ancestral data, our LLS findings were replicated in the Swedish register data (SEDD data) where all the Swedish index persons had available parents, aunts/uncles and grandparent (no missing data). The fact that our conclusions remain identical indicates good performance of the LLS approach.

Implementations

We have more clearly described data availability in the legend of Figure 1 and Table 1 as well as the methods section (line 610-629). In addition, we updated the entire statistics section to clarify our analyses, including the description of all variables included in the models. We further added a more detailed description of replication in the Swedish data to the methods

section and added a discussion of the benefits of replication to the discussion section of the paper (line 616-632 and line 346-349).

Comment I

In supplementary Tables 2 and 3, the units of the labels were (mean), while the results displayed in the tables were proportions; and thus these errors need to be corrected.

Response

We appreciate the reviewer's suggestion. We described in the Table legend that means indicates a proportion for categorical measurements and the group average for continuous measurements. To avoid confusion we will adjust the mean column to mean/prop. and explain in the legend that means represent the average value for continuous variables and proportions are presented for categorical variables.

Implementations

We changed mean into mean/prop. In all applicable Supplementary Tables.

References

1. van den Berg, N. *et al.* Longevity defined as top 10% survivors and beyond is transmitted as a quantitative genetic trait. *Nat. Commun.* **10**, 35 (2019).

2. Berg, N. *et al.* Longevity Relatives Count score identifies heritable longevity carriers and suggests case improvement in genetic studies. *Aging Cell* **19**, 1–15 (2020).

3. Wang, H. *et al.* Global age-sex-specific fertility, mortality, healthy life expectancy (HALE), and population estimates in 204 countries and territories, 1950–2019: a comprehensive demographic analysis for the Global Burden of Disease Study 2019. *Lancet* **396**, 1160–1203 (2020).

4. Pedersen, J. K. *et al.* The Survival of Spouses Marrying Into Longevity-Enriched Families. *Journals Gerontol. Ser. A Biol. Sci. Med. Sci.* **72**, 109–114 (2017).

5. Mourits, R. J. *et al.* Intergenerational transmission of longevity is not affected by other familial factors: evidence from 16,905 Dutch families from Zeeland, 1812-1962. *Hist. Fam.* **25**, 484–526 (2020).

6. Christensen, K., McGue, M., Petersen, I., Jeune, B. & Vaupel, J. W. Exceptional longevity does not result in excessive levels of disability. *Proc. Natl. Acad. Sci.* **105**, 13274–13279 (2008).

Reviewer comments, second round

Reviewer #1 (Remarks to the Author):

This is a revision of manuscript. The authors made significant efforts to address reviewers' criticisms. Significant portion of information about missingness has been added to the revised manuscript.

Reviewer #3 (Remarks to the Author):

The authors made efforts to address my previous comments. I do not have comments.
